# Exploring the Experiences and Challenges of Breastfeeding Beyond 2 Years in the United Kingdom: A Qualitative Study

**DOI:** 10.1111/mcn.70072

**Published:** 2025-08-28

**Authors:** Joelle Morgan, Sara Jones, Amy Brown

**Affiliations:** ^1^ Swansea University Swansea UK

**Keywords:** breastfeeding, extended breastfeeding, infant feeding, motherhood, qualitative research

## Abstract

The World Health Organization recommend that babies are breastfed up to 2 years old and beyond. Breastfeeding beyond infancy continues to provide physical and mental health benefits for mothers and supports nutrition, immunity and development for children. However, there is a dearth of research exploring the experiences of women who breastfeed beyond 2 years, particularly in countries such as the United Kingdom, where only a small percentage of mothers breastfeed past 1 year. This qualitative study explored the experiences of 12 women in the United Kingdom who breastfed or were breastfeeding a child over 2 years old. Semi‐structured interviews were conducted and analysed using thematic analysis, identifying the benefits and challenges of their experiences. Breastfeeding was central to women's parenting style and nurturing of their child, and its impact extended far beyond nutrition. Breastfeeding helped with bonding and soothing and was viewed as central to a gentle parenting philosophy. However, women reported facing barriers such as stigma, especially around breastfeeding an older child in public, disapproval from family and friends, and poor information from healthcare professionals. Despite these challenges, mothers reported a desire to set an example to others and to normalise breastfeeding an older child. When trying to stop breastfeeding, there was a conflict between mothers wanting to be led by their child and a desire to regain their bodily autonomy. These findings reiterate the importance of supporting women to breastfeed for as long as they want to and ensuring that breastfeeding support encompasses infants and children of all ages.

## Summary

- Breastfeeding beyond 2 years was seen as central to a gentle or attachment parenting philosophy and was helpful for bonding and soothing.
- Other motivational factors for breastfeeding beyond 2 years included beliefs around breastmilk's nutritional and immunological benefits and the positive impact of breastfeeding on maternal health and mood.
- Mothers reported significant social stigma and lack of support for longer‐term breastfeeding, but viewed themselves as having a responsibility to normalise it.
- Decisions about when to stop breastfeeding involved complex and competing emotions. Women should be supported to breastfeed for as long as they would like to.

## 1 Introduction

The World Health Organization (WHO) recommends exclusive breastfeeding for the first 6 months of life and continued breastfeeding alongside complementary food until 2 years old and beyond (WHO 2023). Breastfeeding protects infant health, particularly against respiratory and gastrointestinal infections and reduces maternal risk of reproductive cancers, heart disease and diabetes. Supporting women to initiate and maintain breastfeeding is therefore important for public health (Victora et al. 2016).

Research and resources for breastfeeding in the United Kingdom tend to focus on the early weeks and months, with limited attention being given to exploring longer‐term breastfeeding experiences. In part, this is understandable as breastfeeding continuation dramatically declines in the first 6 weeks of life and 80% of women who stopped breastfeeding in the first 6 weeks wanted to continue for longer (McAndrew et al. 2012). As well as the public health impacts, stopping breastfeeding before a woman wants to can negatively impact maternal mental health, leading to feelings of failure, guilt and shame (Brown 2018). Considering this, and that the WHO recommendations are that breastfeeding should continue up to 2 years *and beyond*, support should also be available for longer‐term breastfeeding.

Estimations suggest that fewer than 1% of mothers in the UK breastfeed past 1 year (Victora et al. 2016), though more recent data from the recent Scottish breastfeeding survey report that it may be around 21% (Public Health Scotland 2024). However, recording limitations suggest these figures may be under‐ or overestimated. This is in contrast to data from many regions around the world breastfeeding according to WHO recommendations is much more common: globally, 45% of children are still breastfed at 2 years of age, with the highest rates in African, South American and South Asian regions (UNICEF and WHO 2023). There is no recorded data on breastfeeding beyond 2 years in the United Kingdom. Percentage‐wise, the proportion of women who breastfeed beyond 2 years in the United Kingdom is likely to be very small; however, this may translate to a significant number of people.

Breastfeeding beyond infancy is not a new phenomenon and has been described as being biologically normal and ‘evolutionarily appropriate’ (Dettwyler 2004), but limited research has examined its impact. Breastmilk in longer‐term feeding continues to contain complex immune properties and remains relatively stable in macronutrient content, increasing slightly in energy over time (Mandel et al. 2005; Perrin et al. 2017; Shenker et al. 2020; Czosnykowska‐Łukacka et al. 2018). Previous large meta‐analyses concluded that breastfeeding beyond 12 months continues to protect from infectious diseases, with longer durations associated with a higher IQ and reduced risk of obesity (Sankar et al. 2015; Victora et al. 2016; Lackey et al. 2021). Protective benefits have also been seen for tooth and jaw development (Kobayashi et al. 2010) and supporting nasal breathing (Limeira et al. 2013). Breastfeeding past 1 year also has cumulative effects for maternal health, including reduced reproductive cancer risk (Li et al. 2014) and extended lactational amenorrhoea supporting optimal child spacing (Short et al. 1991).

Limited research in the United Kingdom and similar countries has examined the lived experiences of mothers who breastfeed beyond 6 months (Newman and Williamson 2018) or 12 months (Dowling and Brown 2013; Burton et al. 2022; Jackson and Hallam 2021; Thompson et al. 2020; Säilävaara 2023; Paul et al. 2024), with one exploring the experiences of women who were tandem feeding which included children being fed up to 3 and a half years old (Rodríguez Vázquez et al. 2023). Breastfeeding older babies has been described as a useful parenting tool to soothe upset children or as an aid to bonding (Burton et al. 2022; Jackson and Hallam 2021; Säilävaara 2023; Rodríguez Vázquez et al. 2023; Paul et al. 2024). Prior studies suggest a link between women breastfeeding beyond infancy and attachment parenting (Burton et al. 2022; Green and Groves 2008). Attachment parenting is a philosophy based on forming a strong connection between parent and child, and typically involves prompt responding to cries, co‐sleeping (sharing a sleep space with their child) and baby‐wearing (carrying babies in slings) (Burton et al. 2022). This approach to parenting is thought to have psychological benefits, including better emotional regulation and resilience (Miller and Commons 2010).

However, alongside these positive experiences, social stigma related to longer‐term breastfeeding in the United Kingdom and other Western countries across demographic backgrounds has been documented, and it is often viewed as pointless and strange (Cook 2016; Dowling and Brown 2013; Thompson et al. 2020; Chan and Whitfield 2021; Paul et al. 2024). Stigma stems from prevailing cultural norms that associate breastfeeding primarily with infancy, leading to perceptions that extended breastfeeding is unnecessary or inappropriate (Newman and Williamson 2018). This is reinforced by limited public discourse and media representation, which rarely normalise or support breastfeeding beyond the early months (Jackson and Hallam 2021).

Studies that examine breastfeeding experiences or impacts past infancy tend to have an upper breastfeeding duration of 24 months, particularly in Western regions where only a small percentage of women breastfeed past this time point, meaning in part it is difficult to sufficiently power studies (e.g., Scott et al. 2019; Carletti et al. 2011; Chantry et al. 2006). By having this upper limit, studies therefore fail to capture the different experiences, challenges and barriers women face as their child grows older. For example, in a Canadian study, negative public perceptions were more common in relation to seeing a 2‐and‐a‐half‐year‐old child being breastfed than for a 13‐month‐old (Chan and Whitfield 2021).

The aim of this study was to explore and understand the lived experiences of women practicing breastfeeding beyond 2 years in the United Kingdom, where there is extremely limited data.

## 2 Methods

### 2.1 Study Design and Population

Due to the paucity of literature on this topic, this study followed a simple exploratory qualitative interview research design. Participants were eligible to participate if they were mothers aged 18+, residing in the United Kingdom, who had breastfed at least one child beyond 2 years of age, within the last 5 years, to minimise recall bias. Participants were excluded if they could not speak English, due to no access to translation services.

### 2.2 Recruitment Strategy

Participants were recruited via a purposive sampling approach using social media from one popular global Facebook group called ‘Breastfeeding older babies and beyond’ (59K membership at the time of the research). Permission was sought from the administrator of the group to post an advert on the page. Potential participants self‐selected and were asked to contact the primary researcher via Facebook Messenger or email, where inclusion/exclusion criteria were checked.

### 2.3 Data Collection

Qualitative data were collected through semi‐structured interviews, conducted online via Zoom (*n* = 7) or telephone (*n* = 5), depending on the participant's preference. The interviews were conducted by one researcher (Morgan) to promote consistency. The interviews, lasting between 18 and 54 min, were recorded and then transcribed by Morgan.

The interview schedule (Table 1) was developed by the primary researcher (Morgan), a midwife and mother who breastfed her own children beyond infancy and her supervisor (Jones), an experienced researcher and health visitor and was therefore shaped by their expertise and experiences of the topic. The interview schedule aimed to be exploratory, seeking to simply understand experiences of this under‐researched group; however, it was also informed by the prior literature and hence questions related to stigma (questions 4, 5 and 7), a common experience in longer‐term breastfeeding (Jackson and Hallam 2021), were included. Due to the time‐restraints of the project, the questions were not piloted.

**Table 1:** Semi‐structured interview schedule.

1. Could you tell me about your breastfeeding journey and how old your child was when it came to an end? 2. How long did you think you would initially breastfeed for? 3. Did you decide to stop breastfeeding or did your child make the decision? 4. How did you find breastfeeding in public as your child got older? 5. Did your partner, family or friends have any opinions they shared with you about breastfeeding your child as they got older? 6. What influenced you to continue breastfeeding your child beyond infancy? 7. Did you feel a change in people's or society's views towards you breastfeeding as your child got older? 8. Is there anything that you particularly enjoyed about breastfeeding your child as they got older? 9. Are there any parts that you found challenging or did not enjoy about breastfeeding beyond 2 years of age?

Thematic saturation was monitored throughout the data collection and analysis process; after 12 interviews, we found recurring core themes with minimal new insights emerging. Twelve is an appropriate sample size for qualitative interviews (Creswell and Creswell 2023), with a similar sample size and method being used in previous qualitative research exploring women's experiences of longer‐term breastfeeding (Gross et al. 2017; Newman and Williamson 2018).

### 2.4 Data Analysis

Braun and Clarke's (2006, 2020) thematic analysis framework was used to analyse the data. Through an inductive process of coding and analysis, interview transcripts were repeatedly read to achieve data immersion and look for meanings and patterns. Code development incorporated both semantic and latent meaning. Initially, a semantic approach was used to identify explicit meanings in participants' accounts. As the analysis progressed, a latent approach was also applied to explore underlying ideas, assumptions, and social norms shaping these experiences. Throughout this process Morgan, the primary researcher recorded her thoughts in a reflexive journal. This led to the development of initial codes using NVivo software. Codes were then grouped to identify preliminary themes, which were reviewed to ensure they were meaningful and clearly defined, forming a coherent pattern. A thematic map visually enhanced the analysis, and the themes were compared back to the original transcript to ensure the raw data was represented accurately. Morgan, together with her supervisor Jones, then refined the themes and considered them in relation to each other and the broader narrative.

### 2.5 Ethical Statement

Ethical approval was granted by the Swansea Univeristy School of Health and Social Care Research Ethics Committee. The participants provided their informed consent, and their data were anonymised.

## 3 Results

Twelve participants were individually interviewed in July 2023. Duration of breastfeeding ranged from 2 years and 2 months to 4 years and 11 months. Ten of the 12 participants were still breastfeeding one or more of their children. Due to the limited population of women breastfeeding beyond 2 years, and the method of recruitment via one Facebook group, detailed demographic details were not collected to avoid identifiability.

Six common themes were identified.

### 3.1 Breastfeeding as a Parenting Philosophy

Participants explained how breastfeeding benefited their lifestyle and complimented their parenting philosophy, and that following the child's lead with continuing to breastfeed was important.

It's an easy way for me to parent. Yeah, it's just the easier way for me … if I said no to him it would mean a lot more meltdowns. And you know, we gentle parent as much as we can.

P8

Breastfeeding was also used as a parenting tool by all of the participants and was frequently described as fixing everything. It was used when the child was upset, overstimulated, having a tantrum, to get their child to sleep and to bond or reconnect after periods of separation.

Literally it just it worked for everything. Like if he was upset or crying … Boob. If he was hurt. Boob. Why would I stop doing something that you know, just calms him down in any situation?

P11

It was apparent that breastfeeding was intertwined with the way participants nurtured and comforted their child.

You know when people say like, “Oh, it's just for comfort”? And I think that's so sad, because isn't that just such a valid need for a child? Like why would you not want to provide your child with comfort?

P5

Some felt that the comfort breastfeeding had provided extended even after stopping. One participant described putting her 4‐year‐old to sleep once she had stopped breastfeeding.

At bedtime her comfort thing was to put her hand on my boob. And then after a while she would swap to the other side. I always felt like she was imagining feeding still on one then the other.

P5

Participants reported continuing to breastfeed felt like the right path for them. The opportunity for bonding and reconnecting, particularly after times of separation, was important.

When I went back to work it was always really nice to be able to like have that as our way of being like “Right we're back together now.” This is our thing.

P4

One participant explained that their child has additional learning needs and how important breastfeeding was to receive feedback and love.

I think because of his communication issues … that one thing of us having a little feed at night. He can't say “Love you.” But that's our little bonding time … our little cuddles before bed. And you can just feel the love.

P9

The sentimentality towards their breastfeeding journey was evident. Many women explained how they were newly passionate about breastfeeding as a result of their experiences.

It's like grieving the end of a journey. I feel like that part of mothering my children has ended and that feels really sad and like a momentous thing that people don't really get.

P4

Five of the participants interviewed tandem fed their children as they felt their older child was not ready to stop before their next baby arrived. Breastfeeding was seen as useful for calming both children and managing having more than one child at home.

I felt like I'd kind of unlocked this like, secret hack to … how to get five minutes to yourself. How to get everyone to stop talking anytime I needed a rest. I would plug them on and kind of have a breather.

P5

### 3.2 The Physical Benefits of Breastfeeding

Participants were asked what influenced them to continue breastfeeding beyond 2 years of age. All the participants listed physical benefits for their child, including the nutritional value and milk composition, and the majority described that continuing to breastfeed felt ‘biologically normal’.

The milk changes … I didn't know any of this but the hormones in it from morning to night are different to help drill in the rhythms to your baby … Lipid content as well, things like where their brain has massive leaps it puts way more whey into the milk that's supporting brain development, as opposed to casein that are more for muscle development.

P1

One participant reported the nutritional value of breastmilk as having particular importance as their child is a ‘picky eater’.

It's great nutrition because you know … I've got a picky eater and it's a bit of a problem, especially getting vitamins in. But at least because I'm nursing him, I know he's still getting some form of nutrition from me.

P7

Antibodies being present in breastmilk were also mentioned by every participant and they articulated that breastmilk was the optimal milk tailored to their baby. One participant reported their child had cystic fibrosis which led to continued breastfeeding being even more important.

To be honest, he hasn't really been poorly at all. Like he gets the odd cough, but he's never had to be hospitalised or anything. And I am sure it must be breastfeeding. It must play a part in it … because like it's so clever, isn't it? Like the milk adapts to what they're fighting off. So I just think, why would I ever try and take that away?

P12

The benefits of breastfeeding to the mother's own physical and mental health were also raised as a factor influencing their decision to continue.

It's great for me, you know? Because it's going to help me reduce the chances of X, Y and Z in the future … Like cancer and obesity and all these things.

P2

The positive effect on mood and mental health was reported. This was raised by the two participants who had stopped breastfeeding, both reporting a decline in their mood once they had stopped.

I've always suffered with low mood and things like that. But not once in our breastfeeding journey did I feel low. And then when I stopped … gosh you know when your milk comes in you get the milky blues? You definitely get it when your milk goes out. When I stopped my hormones just plummeted … I was in a hole.

P1

### 3.3 Information Gathering and Support

Many participants stated they did not know how long they would breastfeed for, with most reporting 6 months or a year as their initial plan. Breastfeeding beyond 2 years old was something that developed with time as their knowledge and experience grew. Ten out of the 12 participants quoted the WHO (2023) advice of ‘until two years of age or beyond’ as being influential. It was evident from the participants that they had become well‐informed on breastfeeding, largely developing this knowledge from the internet and social media. This knowledge was gathered to help influence decision‐making and as a response to the perceived social stigma. Social media was used as a source of information by many, providing encouragement to continue and influencing participants' attitudes and subjective norms.

So I initially planned to only breastfeed for six months. But then we got there and we were going really well so I thought I'd try and do a year. And then the breastfeeding groups said the guidance is to do two years or beyond if you can. So then I just kept going … And I think gradually I moved that, erm, that end point in my mind.

P2

I think social media for me has been really useful. Like the people I follow, there's lots of lactation consultants that I've got some good advice from. Like I've not contacted them individually or anything … but just what they are saying on their Instagram.

P3

Seeking advice and information from peers on specialist social media groups for breastfeeding beyond infancy was a common occurrence.

I'm on the global breastfeeding beyond page, which I find very helpful. And I'm so glad it exists because I think it normalises something that women probably feel like, like perhaps they're on their own. There's the most amazing posts that people share, like research and things.

P12

The interviews were conducted in July 2023, with all the children born before July 2021 being eligible. Therefore, many of the women had breastfed during the COVID‐19 pandemic. The use of online breastfeeding support groups, particularly during this time, was beneficial.

I joined a lot of Facebook breastfeeding groups because obviously in person ones weren't possible with COVID. Yeah … and that the online ones were great. I could just have a squirrel in the middle of the night and just read other people's stories and advice.

P10

### 3.4 Social Responsibility

Participants described how they felt solidarity with other women who were also breastfeeding older children and how they were seen as positive role models.

Anybody else who'd kind of breastfed for like a longer amount of time would be like, “Oh my God, I breastfed mine until they were four.” And, you know, like there was this instant bond.

P5

She gave me so much courage to carry on because she I knew she was from like the same village as me and, and I just thought, well, if she's doing it, why, why aren't I?

P9

However, in general society, it was often not discussed, or the topic was avoided.

It just makes me think of that old film Fight Club, everybody knows there's Fight Club … But you don't talk about it. Like everybody knows that there's, you know, a mum out there that's breastfeeding past two, but you can't talk about it.

P11

All of the participants discussed the need or the desire to ‘normalise’ breastfeeding older children. This was often presented as a perceived responsibility to make breastfeeding an older child more visible.

I feel like I've got a duty in a way, to make it visible and help educate people for the future … like to help women coming through behind me.

P8

There were differing experiences of support from family and friends amongst the participants. Many women reported judgement from their family and pressure to stop breastfeeding.

My family are like “Oh you're still feeding him” and I remind them again about the advice and guidance and say “You can look it on the Internet. A lot of things have changed since you know, we were kids.”

P2

The influence a woman's partner had on her decision to continue feeding was discussed. The majority reported their partner was supportive, but one participant reported having to conceal breastfeeding from their partner, who did not approve.

Like he didn't actually know I was still feeding my son when my daughter arrived because he was like you need to stop now. So I just went “Yeah, OK … no worries.” And I just … I'm always up before him, so I just used to still feed him in the morning. I think I carried on feeding him for another four months after that.

P10

### 3.5 Social Stigma

Many of the participants reported they noticed a change in society's support for breastfeeding around the age of one. Participants were being questioned by colleagues, family or friends.

When he was nearing one, I started getting a lot more questions. “When are you gonna stop feeding him? Why are you still feeding him?” Questions like that.

P7

The participants also described judgemental or ill‐informed comments coming from healthcare professionals assuming women had stopped breastfeeding, or coercing mothers into stopping.

Yeah, my health visitor told me how I'd still be feeding him at the school gate and how he'd be asking me for bitty in reference to, you know, Little Britain.

P9

You go to the doctors, even if your child is only one. They'll prescribe something and I'll say “Oh, is that safe for breastfeeding?” And they've gotta look online. And my niece … she was refused antibiotics. She had septic tonsillitis. I mean, she could barely swallow. And the GP wouldn't give her antibiotics unless she stopped breastfeeding.

P9

Many mothers said people assumed they had stopped, and the shock when people found out led them to hide the fact that they were still breastfeeding.

It's definitely an assumption that we're not still feeding, and it's when they find out, it's the shock or surprise.

P3

The participants were asked about breastfeeding in public beyond the age of two. Most participants said their child usually only fed when they woke up or before bed, so the need did not frequently arise. However, some also reported avoiding breastfeeding in public due to the social stigma.

I do tend to avoid it if I can or distract him. Honestly … it is the stigma around it. And also, he's older so I can tell him “Look, we'll have some when we get home” or I make him wait until we're back at the car.

P8

It was perceived that a child's autonomy led to the stigma around continuing to breastfeed. It was suggested that when their child could walk, talk or physically demand a breastfeed that society was less approving of it.

Once they're sort of old enough to ask for it, then they then they're too old to have it anymore. That's what people think…

P10

He will physically like pull my top down to get to my boobs now. And there was a phase where he used to say “Two boobies,” and want both out at the same time. Obviously, that's not something I would want to do in public.

P3

Many of the participants described how they previously found breastfeeding an older child strange, but now that they were doing the behaviour themselves, they felt conditioned to find it normal.

I think I always thought it was a little bit weird when people fed older children. But then the longer I did it for, the more I thought how natural it was. But then weirdly … in between my two children, I remember seeing people feeding an older child and that weird feeling came back … I think it was like a lack of exposure to it.

P4

One participant described breastfeeding their toddler felt like a ‘dirty little secret’. Another participant described how it felt to conceal her breastfeeding from her partner.

I feel like really sneaky doing it. And I was worried he'd give the game away. But I was just like, well if I don't wanna give up, and he doesn't wanna give up … Like, that's up to us, really. It's not really up to you.

P10

### 3.6 Control When Breastfeeding Older Children

Many participants reported the lack of bodily autonomy when asked if they found breastfeeding beyond 2 years old challenging due to their child's physical capabilities, which led to breastfeeding not being a relaxing experience anymore.

I think it's really taxing on your body. Like, it's all consuming. Even when you're not doing it very often, it's still like a really bodily experience. And then when we finally stopped I was relieved because I had wanted that kind of … like my body back.

P5

So with my youngest I'd been getting tired of her twerking on my lap feeding. And just like the way they kind of move around and can't sit still while they're feeding. There isn't anything relaxing about it now.

P4

They also described implementing boundaries to mitigate these challenges on when or how long their child could feed.

As he's got a bit older now, I've definitely had to set more boundaries with nursing. So it will be “We'll play one song and you can nurse for the song, and then you've got to come off.” I had to kind of develop tactics to try and get him off.

P8

Some of the participants were waiting or had waited until their child decided to stop breastfeeding. However, the process of mothers choosing to stop was also described by many participants.

I started introducing the idea a good couple of months before, actually. And then just started cutting down, like to every other day. Then every third day.

P10

Participants also described how difficult it was to stop breastfeeding before their child was ready, and how they struggled to find support in stopping.

You know, dealing with trying to tell them it's not time, or it's not there is hard when they can actually see your boobs, can't they? It's not like, with a dummy or bottles you can put them in the bin. You can't put your boobs in the bin.

P9

People don't understand it until they've done it. And everyone's like “Well, it's easy to just stop, isn't it?” And it's … it's really not just easy to stop breastfeeding. There's a big misconception about just stopping one day, and that's it.

P7

Many women explained the lengthy process involved in preparing their child to stop, including reading books and telling stories about stopping breastfeeding and slowly reducing the practice, akin to breaking a habit. As previously described, women used breastfeeding to calm their children in line with gentle or attachment parenting, therefore, some noted that stopping a breastfeeding episode when the mother was ready was often more difficult than it might have been dealing with the initial upset child.

Some reported they felt aversion towards feeding their older child. This is often described as an overwhelming feeling of agitation, wanting to unlatch the child and a ‘skin‐crawling’ sensation.

It feels like my skin is crawling and like … I just feel like I literally wanna push his face off me. It's the most horrible feeling ever. You know, unless you've had aversion, you just honestly can't understand how horrible the feeling is.

P7

## 4 Discussion

Our findings highlight the complex experiences of women who continue to breastfeed a child past 2 years old. Mothers described positive benefits, particularly in terms of bonding and connection with their child, and subsequent impacts upon wellbeing. Within this motivated and ‘successful’ sample, mothers had been able to identify different sources of support to enable them to continue feeding, but also felt stigma from others, or an assumption that breastfeeding had ended. Given the recommendations that breastfeeding continue for as long as mother and baby are happy, the findings are important in helping us to understand the support needs of this small but growing percentage of women in the United Kingdom who breastfeed through infancy and beyond.

### 4.1 The Benefits of Extended Breastfeeding for Mothers and Older Children

Mothers described several benefits to themselves or their child in continuing to breastfeed past 2 years old. Breastfeeding was seen as a way of continuing to connect with and bond with their child and was part of a broader mothering identity, reflecting themes in previous research with older infants breastfed past the first year (Green and Groves 2008; Faircloth 2009; Brown 2018; Burton et al. 2022). Indeed, this connection is echoed in the only child participant study of breastfeeding past infancy, where children's descriptions of how breastfeeding made them feel included ‘feel good, feel happy’, ‘loved’, ‘cuddly’ and ‘close to mummy, mummy loves me’ (Gribble 2008, 1072).

It also remained a way to calm or soothe an older baby or child beyond infancy. Breastfeeding releases oxytocin in both babies and mothers, reducing blood pressure, lowering cortisol (stress hormone levels) and promoting feelings of relaxation (Uvnäs‐Moberg et al. 2020). Skin‐to‐skin contact when feeding calms and soothes babies and may foster children's later emotional and behavioural development (Rheinheimer et al. 2022, 2023). Continued breastfeeding also supported maternal mental health. Like in babies, oxytocin release and skin‐to‐skin contact are also thought to have calming effects on mothers' neurophysiology and have been associated with a lower risk of maternal depression and anxiety (Stuebe et al. 2013; Thul et al. 2020; Cooijmans et al. 2022). Furthermore, the experience of being able to continue breastfeeding, the comfort and connection that it brings, and the pride at being able to meet one's goals may support maternal wellbeing (Brown 2018).

The nutritional value of breast milk beyond infancy was also highlighted as a motivating factor to continue. The 2008 FITS study of over 1400 children aged between 24 and 47.9 months, found that milk (breast/formula or cow's) continued to provide around 20% of children's energy requirements (Reidy et al. 2017) and another 2018 study revealed that human milk composition changes with extending feeding increasing in its fat and protein and energy content in samples of milk from mothers breastfeeding beyond 2 years, to meet the needs of the growing child (Czosnykowska‐Łukacka et al. 2018). However, this and other older studies of breastmilk composition beyond 1 year (Mandel et al. 2005; Perrin et al. 2017) are limited by small sample sizes. The limited evidence base supporting the nutritional benefits of breastmilk in the diets of toddlers may contribute to beliefs among healthcare professionals that it is of little nutritional value and ‘like water’ seen in both research (Zhuang et al. 2020; Jackson and Hallam 2019) and media reports (BBC News 2018). This, combined with aggressive marketing of infant formula, which normalises the need for follow‐on or toddler milks, can be strongly influential on mothers' decision making (Paul et al. 2024; Richter et al. 2024).

### 4.2 Challenging Attitudes Towards Breastfeeding an Older Child

The effect of healthcare professional support on breastfeeding beyond infancy was explored by Jackson and Hallam (2019), where participants noted a marked shift in healthcare professionals' attitudes towards supporting breastfeeding after the age of one. Healthcare professionals' language and other subtle responses can either empower mothers to continue or cause self‐doubt and a lack of belief (Jackson and Hallam 2019). In our study, misconceptions around breastfeeding and medication use were mentioned, for example, one participant was denied antibiotics by the GP because of breastfeeding, echoing previous findings on this issue (Jones and Breward 2010; Brown et al. 2019). Research has found that GPs have limited knowledge of breastfeeding and medical students are inadequately prepared to support breastfeeding mothers in their training (Biggs et al. 2020). Participants in this study self‐identified as strong‐willed and said that they had a response prepared if they were questioned on their breastfeeding behaviours by healthcare professionals, friends, family or members of the public.

Most of the mothers in our study experienced social stigma, which worsened as their child got older, leading to some feeding secretly, even hiding it from their partner, family and friends and avoiding breastfeeding in public. This finding aligned with that of other studies of longer‐term breastfeeding in the United Kingdom and Ireland (Dowling and Brown 2013; Thompson et al. 2020; Jackson and Hallam 2021; Paul et al. 2024). Some women who continued to breastfeed in public beyond 2 years of age explained how they did it more discreetly than when their child was younger, such as using their partner for support in hiding the behaviour. Women in previous studies also reported limiting breastfeeding to the home when their child was older (Dowling and Pontin 2017; Stearns 2011). Other mothers in our study explained that they did not actively avoid breastfeeding outside the home, but that it simply did not happen because their child only fed at bedtime now.

Negative attitudes towards breastfeeding in public have long been identified as a barrier to breastfeeding (Grant 2016; Morris et al. 2020; Bresnahan et al. 2020) and are not limited to breastfeeding older babies, though attitudes are more likely to be negative as the child gets older (Chan and Whitfield 2021). Social media analyses of public attitudes towards breastfeeding in public have found disgust towards breastfeeding women to be commonplace, with women often accused of being exhibitionists, exposing themselves for sexual or political reasons (Grant 2016; Bresnahan et al. 2020). Media coverage of breastfeeding older children is often negative. In 2021, a Time magazine cover featuring a mum, Jamie Lynne Grumet, breastfeeding her 3‐year‐old son ignited significant backlash and was called ‘exploitative and extreme’ or akin to ‘child molestation’ (Braiker 2012).

However, despite the prevailing perception of breastfeeding older children being socially deviant, many mothers in our study, rather than feeling shame, reported a sense of responsibility to act as a role model in their community in line with findings of previous studies of longer‐term breastfeeding (Newman and Williamson 2018; Thompson et al. 2020). Such role‐modelling has been termed ‘positive deviance’. In health promotion theory, positive deviants practice less common but healthy behaviours and can influence others (Rose and McCullough 2017). Positive deviant role modelling has been successfully used in relation to breastfeeding initiation and continuation (Ma and Magnus 2012; Siraneh et al. 2021). Positive representation of breastfeeding in the media, such as TV and film, is also likely to be useful in tackling societal stigma (Brown 2016). The media is a source of health information, influencing people's constructed norms and therefore perceptions of breastfeeding beyond infancy. While media influence was not specifically highlighted by participants in this study, participants in Newman and Williamson's (2018) study described the desire to see more older children being breastfed on television shows.

### 4.3 Navigating Transitions

Women in our study described the complexity of navigating the transition to stopping breastfeeding, as also seen in previous research (Burton et al. 2022). Lunau (2016) describes the process of stopping breastfeeding, advising that some children stop abruptly, but for many it is a gradual process of cutting down feeds, so the child emotionally adjusts, and the mother's milk supply steadily reduces. Some women experienced breastfeeding aversion, which is also a common phenomenon (Morns et al. 2021; Yate 2017), particularly in neurodivergent women (Grant et al. 2022). Women described the internal conflict they experienced with wanting to breastfeed their child, whilst having irrational urges to push their child off the breast. Breastfeeding aversion is an under‐researched area, and experiences referred to in this current study add to the limited body of evidence.

### 4.4 Online Support

Online support groups were a source of support for women, especially during the pandemic, by normalising the behaviour and increasing women's self‐efficacy. Online breastfeeding support groups have been well utilised by many mothers for the last decade, providing a valuable sense of community and advice based on lived experiences, which is often deemed more beneficial than advice from healthcare professionals (Black et al. 2020; Clapton‐Caputo et al. 2021; Morse and Brown 2022). However, advice and support accessed online is mainly offered by volunteers, therefore raising the important issue of regulation and credibility of the information provided. The value of specialist support groups for breastfeeding older children was raised by participants in our study and previous literature, as women report feeling stigmatised when discussing feeding older children in the usual breastfeeding support groups, which tend to be aimed at younger infants (Burton et al. 2022; Newman and Williamson 2018; Thompson et al. 2020).

### 4.5 Strengths and Limitations

Interviews were undertaken using the Zoom platform, and this was seen as essential for allowing flexibility for participants who were busy mothers of young children. A further advantage is that participants were able to be comfortable in their own homes, promoting familiarity and potentially lessening social desirability effects (Keen et al. 2022). However, compared with in‐person interviews, online interviews reduce the availability of non‐verbal cues, which may be important for contextualising answers and establishing rapport (Tomás and Bidet 2024). Digital exclusion may also be a limitation; even though participants in our study were active on Facebook, video interviewing relies upon having a good WiFi connection or sufficient data allowance.

Due to the niche population and the stigmatised nature of breastfeeding older children, it can be difficult to access women breastfeeding older children to capture their experiences. This study used breastfeeding support groups on social media to successfully recruit participants; an approach frequently deployed in breastfeeding research; however, self‐selecting participation has its limitations. Participants' views about social media use might introduce bias as those women breastfeeding beyond 2 years of age and not finding online support groups beneficial may not have engaged with the research. Also, mothers in this study who continued to breastfeed beyond 2 years of age appeared to be confident and well educated about their choice. This may have resulted in them being keen to participate and explain their perceived benefits of breastfeeding, to promote and normalise the behaviour. Furthermore, this sampling strategy cannot ensure that a fully diverse spectrum of cultural perspectives is captured, potentially constraining the representation of the range of experiences associated with longer‐term breastfeeding in the United Kingdom.

In the participant information and interviews, the primary researcher (Morgan) was transparent by stating their background as a midwife. Being a midwife may have facilitated access to potential participants who might possibly be willing to share experiences with a researcher who is a knowledgeable professional (Berger 2015). However, this may have altered the power dynamics of the interviews, and participants may have withheld information they felt they might be judged for, such as co‐sleeping or other controversial topics.

The primary researcher also breastfed their own child beyond infancy, positioning herself as somewhat of an ‘insider’ (Berger 2015). Insider status can also be positive; having shared experiences of breastfeeding beyond infancy may have enhanced the researcher's ability to understand implied content and nuances used by the participants. On the contrary, it is acknowledged that the participants may have withheld information that they assumed was obvious to the researcher. Being reflexive of their position by keeping a journal and using open‐ended questions and probing to expand on points helped the researcher gain rich, detailed responses and minimise bias as far as possible. Similarly, while analysing the data, reflexive journaling allowed the primary researcher to consider the influence of their own experiences, especially for latent interpretation. Regular supervisory discussions were also important to minimise interpretation bias. The supervisor (Jones)—who does not have personal experience with breastfeeding—provided a contrasting perspective, which served to challenge assumptions and interpretations that may have been influenced by the researcher's own experiences. This dynamic helped maintain analytic balance and encouraged ongoing critical reflection.

## 5 Conclusion and Future Directions

This qualitative study adds to the limited literature on breastfeeding beyond 2 years of age in the United Kingdom. It has provided rich data surrounding women's experiences, the perceived benefits of longer‐term breastfeeding and the challenges they face. Breastfeeding was considered to be central to women's parenting style and nurturing of their child, and the impact of it extended far beyond nutrition. It is therefore vital that women are supported to breastfeed for as long as they would like to. Health professionals who come into contact with breastfeeding mothers should be aware of the WHO recommendations and the ongoing benefits of breastfeeding beyond infancy. Specifically, more support is needed for prescribers to confidently treat breastfeeding mothers without them needing to stop. The Breastfeeding Network's ‘Drugs in Breastmilk’ factsheets are a useful resource (The Breastfeeding Network n.d.), as is Brown and Jones (2019) book. Wider societal normalisation of breastfeeding beyond infancy requires a significant cultural shift, which is especially difficult given the small numbers of women reaching this breastfeeding duration. More supportive portrayals of breastfeeding in media and public discourse could play a valuable role in reshaping perceptions.

Further research may wish to explore the relationship between longer‐term breastfeeding and maternal mental health, as well as research developing interventions targeted at changing healthcare professionals' knowledge and attitudes towards breastfeeding older children, including whether medication can be taken while breastfeeding. Future work should also explore the cultural diversity of experiences in women who practice longer‐term breastfeeding in the United Kingdom. Studies exploring the representation of breastfeeding older children in the media would also be useful in understanding societal attitudes and moving towards normalising the behaviour.

## Author Contributions

**Joelle Morgan:** conceptualisation (lead), investigation (lead), formal analysis (lead), project administration (lead), writing – original draft preparation (lead), writing – review and editing (supporting). **Sara Jones:** supervision (lead), conceptualisation (lead), investigation (lead), formal analysis (supporting), writing – original draft preparation (supporting), writing – review and editing (lead). **Amy Brown:** writing – original draft preparation (supporting), writing – review and editing (supporting).

## Conflicts of Interest

The authors declare no conflicts of interest.

## Data Availability

The data that support the findings of this study are available on request from the corresponding author. The data are not publicly available due to privacy or ethical restrictions.
